# Traditional Islam in Kazakhstan: historical formation, state discourse, and contemporary challenges

**DOI:** 10.3389/fsoc.2026.1786903

**Published:** 2026-05-08

**Authors:** Shamshadin Kerim, Maxat Kurmanaliyev

**Affiliations:** Egyptian University of Islamic Culture Nur-Mubarak, Almaty, Kazakhstan

**Keywords:** extremism, Kazakhstan, policy, religion, state, traditional Islam

## Abstract

This paper examines notion, historical development, and contemporary trajectories of “Traditional Islam” in Kazakhstan, and places it in the context of the broader discussions on religion, state governance, and national identity in post-Soviet societies. It argues that Traditional Islam in Kazakhstan is not just a religious category, but a complex phenomenon shaped by Hanafi jurisprudence, Maturidi theology, Sufi traditions, local customs, and state regulation. The article shows how the notion has been shaped historically through changes in the culturally embedded pre-modern Islam, the Soviet secularization, and finally the post-1991 institutionalization and state support period. By comparing the situation in Kazakhstan with the experiences of other countries, such as Russia, Morocco, Indonesia, and Western Europe, the paper demonstrates that “Traditional Islam” is being utilized as a state-authorized discourse for ensuring social stability, political loyalty, and cultural continuity. The study calls for a more accurate conceptualization and warns against oversimplifying religious diversity by relating it only to security concerns. The key finding demonstrates that “Traditional Islam” in Kazakhstan functions as a state-mediated framework designed to regulate the religious sphere, ensure social stability, and reinforce national identity.

## 1 Introduction

Religion studies in Central Asia have been the subject of growing academic interest over the last few decades, mainly as a result of rapid social changes, the nation-building processes of the post-Soviet era, and the reshaping of religious identities in the public sphere. Within this context, Traditional Islam in Kazakhstan has been identified as one of the most complicated and controversial issues whose academic study has been accompanied by a certain degree of anxiety about, among others, its conceptual limits, its historical development, and its possible paths of evolution. Since the period that Kazakhstan is continuously branding itself as a secular state with religious tolerance, the question of the nature and role of Traditional Islam has become a matter of concern for scholars.

Traditional Islam in Kazakhstan is recognized as a historically rooted form of Sunni Islam, mainly linked to the Hanafi school of jurisprudence and being under the influence of local customs, Sufi practices, and pre-Islamic cultural elements. But, the lack of a clear-cut definition has caused the matter of ongoing debates among the academic community (Laruelle, 2018). Such conceptual uncertainty is particularly significant in the post-Soviet period, when religious revival intersected with the emergence of transnational Islamic movements and alternative interpretations. The scholars pointed out that if we don't take a fine-grained approach to Traditional Islam, analytical frameworks might unintentionally simplify religious dynamics or equate indigenous practices with imported ideologies (Montgomery, 2007).

In addition to the existing scholarship, several important studies further contextualize Islam and state-building in Central Asia. (DeWeese and Gross 2018) and (DeWeese 2023) highlights the historical role of Sufi networks and religious authority in Central Eurasia, demonstrating the deep-rooted and socially embedded nature of Islamic traditions in the region. (Tasar 2017) provides a detailed account of the institutionalization of Islam under Soviet rule, showing how religious structures were reshaped but not eliminated by state policies. From a political and sociological perspective, (Sharipova 2018, 2020) analyzes state-building processes and the role of informal institutions and national identity in Kazakhstan, offering a crucial framework for understanding state–religion relations. Earlier works by (Diuk and Karatnycky 1990, 1993) situate these developments within broader post-Soviet transformations, emphasizing the role of identity, society, and political change in newly independent states. Together, these studies strengthen the analytical foundation for examining the evolution and contemporary construction of Traditional Islam in Kazakhstan.

The development of traditional Islam in Kazakhstan is closely linked to a series of historical transformations in the region. These include the Islamization of the Kazakh steppe from the eighth to the 18th centuries, the influence of Sufi networks, and the integration of Islamic norms within nomadic social structures. Historically, Islam in Kazakhstan was less of a formal legal system and more of a flexible moral and cultural code deeply ingrained in everyday life, according to studies of Islam in Kazakhstan (Privratsky, 2001). Under the Soviet regime, religious practices were almost entirely banned, which led to a break-up of religious knowledge and a turning inward of faith. This upheaval still influences the way religion is experienced today and, according to some researchers, leads to a type of “cultural” or “folk” Islam rather than a strictly doctrinal one (Keller, 2007).

Since its independence, Kazakhstan has always been promoting Traditional Islam as a unifying factor and countermeasure to religious extremism. The state-led support of such a policy has even increased scholars' attention, as they seek to clarify if Traditional Islam was the natural way of following historical practice or a newly invented version under the influence of politics and security issues (Schatz, 2014). The future of Traditional Islam is thus closely linked to the main topics of religious leadership, faith of young people, and globalization.

Despite a growing body of literature, gaps remain in systematically analyzing Traditional Islam as a dynamic concept that evolves across historical stages while responding to contemporary challenges. Addressing these gaps is crucial not only for advancing academic debates but also for informing evidence-based approaches to religious policy and social cohesion in Kazakhstan. This study seeks to contribute to this discussion by examining the concept, formation stages, and future perspectives of Traditional Islam in Kazakhstan within a comprehensive analytical framework.

Building on these debates, this study approaches “Traditional Islam” not merely as a theological category but as a politically mediated construct. To demonstrate this, the study operationalizes the concept of a “state-mediated construct” through three observable dimensions:

- institutional control over religious authority,
- discursive framing in official narratives, and
- regulatory differentiation between “traditional” and “non-traditional” Islam.

These dimensions guide the empirical analysis and allow for a systematic examination of how “Traditional Islam” is constructed and maintained in Kazakhstan. By addressing these issues this paper contributes to the literature by providing a systematic, mechanism-based analysis of Traditional Islam.

In addition, this article refers to the work of Talal Asad and Pierre Bourdieu. (Asad 1993, 2003) conceptualizes religion not as a fixed or universal category but as one shaped through historical processes, power relations, and institutional regulation. It is particularly relevant for understanding how “Traditional Islam” in Kazakhstan is constructed through state discourse and governance rather than derived solely from theological tradition. As for Bourdieu's concept of symbolic power and the religious field (Bourdieu, 1986, 1990), it provides a framework for analyzing how state-aligned institutions define and legitimize religious authority while marginalizing alternative interpretations. Together, they enable this study to interpret “Traditional Islam” as a product of institutional power and discursive construction, thereby moving beyond purely descriptive or theological accounts.

Together, they enable this study to interpret “Traditional Islam” as a product of institutional power and discursive construction, thereby moving beyond purely descriptive or theological accounts.

The Kazakhstani case reflects a broader pattern. (Roy 2004) describes how modern states promote “domesticated” forms of Islam, while (Cesari 2013) underscores the role of state institutions in defining acceptable religious expression in relation to governance and security.

This aligns with broader debates on religious authority and modern governance, as scholars such as Saba Mahmood shows how religious traditions are reshaped through institutional power and normative regulation (Mahmood, 2005).

## 2 Research methods

This research combines various qualitative methods and draws on different disciplines to accomplish the goals set. The focal point was local Islam in Kazakhstan, but to understand it broadly, the authors compared local case studies with large-scale theoretical models of political sociology and also used discourse analysis. Besides, as the term itself was unclear and vague, it was very important that the authors used various methods so that they may show not only the formation of the concept but also its present-day institutional and political sides. First, historical-analytical methods were used to trace the long-term development of Islam in the Kazakh steppe, focusing on the Islamization process between the eighth and eighteenth centuries, the role of Sufi networks, and the interaction between Islamic norms and nomadic social structures. Secondary historical sources were critically reviewed to assess how Islam functioned as an ethical and cultural framework rather than a rigid legal order. Second, the study applied textual and discourse analysis to academic literature, policy documents, official religious publications, and media materials produced by Spiritual Administration of Muslims of Kazakhstan (SAMK), to identify dominant narratives and normative assumptions in official definitions of Traditional Islam. Third, comparative analysis situates the Kazakhstani case within a broader international framework, examining examples from Russia, Morocco, Egypt, Indonesia, and selected Western European countries to identify common patterns and differences in how states construct and promote “traditional” or “moderate” Islam. Fourth, the study utilized content analysis of digital and media outputs, including sermons, articles, and social media materials disseminated by official Islamic institutions. The analysis focused on recurring themes such as moderation, harmony between Islam and national traditions, loyalty to the secular state, and the rejection of extremism. Finally, the study utilized a conceptual-theoretical framework as a means of responding to the deficiency of a clear definition of Traditional Islam in the academic works of Kazakhstan. Through a combination of perspectives from Islamic studies, sociology of religion, and political theory, the research develops a tentative definition of Traditional Islam as a historical, cultural, and institutional manifestation of Sunni Islam. The mix of methods here makes the analysis both rigorous and harmonious with the actual religious experience of Kazakh society.

## 3 Results and discussion

### 3.1 Religious, social, and political contexts of the evolution of Traditional Islam in Kazakhstan

The evolution of Traditional Islam in Kazakhstan was influenced by various religious, social, and political factors that changed over the course of several centuries. In the Kazakh context, Islam did not develop into a single or purely doctrinal system. Instead, it evolved as a flexible and adaptive tradition shaped first by nomadic culture, then by various forms of imperial governance, followed by Soviet secularization, and finally by post-independence state-building. Grasping these factors is crucial for the explanation of both the unique nature of Traditional Islam in Kazakhstan and its present-day relevance.

In terms of the theology, Traditional Islam in Kazakhstan is mainly based on Sunni Islam of the Hanafi school which by its nature allowed for an interpretative freedom and accommodation of local customs. Islam gradually and unevenly spread across the Kazakh steppe from the eighth to the 18th century and this spreading was facilitated mostly by Sufi preachers rather than formal religious authority institutions (DeWeese, 1994). Traditionally Islam was shaped just as much by social factors. The nomadic lifestyle of the Kazakh people had a huge influence on the religious organization, making it impossible to develop highly centralized religious institutions such as madrasas or formal clerical hierarchies. The main channels for religious knowledge at that time were therefore, among others, informal networks, elders, and local spiritual figures. The decentralized structure of this religion was able to serve as a moral compass, thus leading to social cohesion, customary law (adat), and tribal identity (Keller, 2007).

The Soviet period (1917-1991) deeply changed religious life as the authorities introduced state atheism, closed religious education, and even abolished the clergy's support. Nevertheless, the state policies only succeeded in pushing Islam to a private and cultural level instead of wiping it out altogether. Experts refer to this period as one of “religious minimalism,” when Islamic identity survived mainly through the performance of rituals like funerals, circumcision, and the celebration of holidays, although the Islamic institution was largely removed (Laruelle, 2018). Such a situation has had a great impact on the religious revival of the post-Soviet period as most Kazakhs came to Islam again through cultural memory rather than through religious studies.

Political factors have played a major role in the development of the present-day concept of Traditional Islam in Kazakhstan. Upon gaining independence in 1991, the Kazakhstani authorities vigorously supported Traditional Islam for several reasons. First, institutional regulation: the state empowered the Spiritual Administration of Muslims of Kazakhstan (SAMK) to oversee religious life and define legitimate Islamic practice, effectively centralizing theological authority within a state-aligned structure. Second, discursive construction: official narratives consistently present Traditional Islam as a form of religion characterized by moderation, interfaith tolerance, and loyalty to the secular state, aligning religious legitimacy with political values. Third, boundary-making: the promotion of Traditional Islam is explicitly linked to the need to counter “extremism” and “foreign” religious movements, thereby constructing a binary between acceptable and unacceptable forms of Islam. All these elements demonstrate that the concept functions not merely as a reflection of historical religious practice, but as a structured instrument of governance (Omelicheva, 2015). While Traditional Islam is often described in theological terms—such as adherence to Hanafi jurisprudence and Maturidi theology, its contemporary meaning in Kazakhstan cannot be reduced to doctrinal features alone. The formal definition promoted by SAMK and supported by state institutions explicitly incorporates political criteria, including loyalty to the secular state and rejection of non-approved movements (Schatz, 2014).

Though Islamic ritual practices based on local tradition were present in Kazakhstan for hundreds of years, the expression “Traditional Islam” was not widely used scholarly or publicly until circa 2010. Before this time, religious conversations were more likely to recognize Islam historically, culturally, or geographically but rarely with a uniform concept label. The active use of this term matched the rise in public and state worries about the danger of religious radicalization as well as the exposure of foreign Islamic movements in the former Soviet area (Laruelle, 2014).

Nowadays, the idea of Traditional Islam is being compared with the versions of Islam that came about as a reaction to the modern and globalized world. Such a division raises question of authenticity of the Islam as Salafi movement argue its current interpretation do not meet initial teachings of the Quran and Sunnah. Salafis advocate for pure Islam and claim modernist adaptations of Islam should be rejected as they are considered to be corruptions and deviations from the right path (Ekaterina, 2016). Besides pointing to the Muslim community's internal doctrinal diversity, these debates also reflect wider tensions between tradition and modernity, urging scholars to undertake an inquiry into what Traditional Islam really means and how significant it is in the present time (Nasr, 2011) as well as to explore its impact on today's Muslim identities. Tradition and modernity are intertwined in such a way that their interaction determines the discourse on the future of Islamic thought and practice.

### 3.2 Defining “Traditional Islam” in Kazakhstan

Since Kazakhstan's independence in 1991, the notion of “traditional Islam” has emerged as a central concept in discussions of religion, national identity, and state security. Traditional Islam in Kazakhstan is a historically rooted, culturally embedded, and politically mediated expression of Sunni Islam, reflecting, to a certain extent, the state's delineation of religious freedom boundaries in a secular, post-Soviet context (Khalid, 2007).

In the context of Kazakhstan, traditional Islam is regarded as a variant of Sunni Islam which relies on the Hanafi school of jurisprudence. It is also characterized by the incorporation of local customs and the impact of Sufi mysticism. Traditionally, this style of Islam evolved among the nomadic and semi-nomadic Kazakh people whose religious life was closely intertwined with social morals, kinship groups, and cultural festivities rather than being regulated by formal legal bodies (Schatz, 2004).

“Traditional Islam” in Kazakhstan is commonly associated with Hanafi jurisprudence and Maturidi theology, but these features do not distinguish it from other Sunni traditions. Its specificity lies in its historical embedding within a nomadic social context, where Islam functioned as a flexible moral and cultural framework closely linked to customary law (adat) and kinship structures (Alzamova and Akhunov, 2021). In the post-Soviet period, this form of Islam has been reinterpreted and institutionalized through state structures, particularly the Spiritual Administration of Muslims of Kazakhstan (SAMK). As a result, “Traditional Islam” represents a context-specific configuration shaped by cultural heritage, Soviet secularization, and contemporary state governance (Laruelle, 2018).

Nevertheless, today the term has acquired formal and political significance. The Spiritual Administration of Muslims of Kazakhstan defines traditional Islam as Sunni Islam of the Hanafi madhhab that aligns with national traditions and shows loyalty to the secular state. This definition is widely supported by state authorities and used to distinguish accepted religious practice from movements labeled as foreign, radical, or extremist (Yemelianova, 2010).

In terms of doctrine, Traditional Islam in Kazakhstan is deeply rooted in the Sunni Hanafi jurisprudence, which dominated in the Central Asia over the centuries. It is the methodological flexibility of the Hanafi school that, among other things, a handful of istihsan (juristic preference) and acknowledgment of urf (custom), have made it such a great fit to the nomadic and tribal situations, so that the Islamic norms could be enough together with the local practices (Saifullin et al., 2026). Theologically, Traditional Islam in Kazakhstan is generally associated with Maturidi theology, which emphasizes reason, balance, and moderation in matters of faith. The emphasis on moderation and rationality is rooted in the Hanafi tradition, particularly in Abu Hanifa's flexible and context-sensitive approach to Islamic thought, which continues to shape contemporary interpretations of “moderate” Islam (Bulan et al., 2025). This orientation is often contrasted with more literalist or scripturalist approaches, which are frequently labeled “non-traditional” in official discourse (Laruelle, 2011). In his article Smanov claims that Shari'a was selectively integrated into judicial practice, mainly in family, inheritance, and ethical matters, while adat dominated procedural rules and everyday governance. The study challenges the dichotomy between Islamic law and customary law by showing their pragmatic coexistence and mutual adaptation within the Kazakh legal tradition (Smanov et al., 2025).

On a cultural level, Traditional Islam in Kazakhstan has been tightly linked with the Kazakh adat or customary law. In the past, religious life was comprised of reverence for saints, pilgrimage to local shrines, and rituals honoring ancestors these were the practices which community identity and moral values were reinforced through, rather than strict legal observance (Schatz, 2004). Sufi decorations, especially those related to Ahmad Yasawi and Yasawi-derived spirituality, were main factors in shaping people's religion. Even though official Sufi establishments were weakened under the Russian imperial and Soviet eras, Sufi morals and signs continued to be part of the local religious culture (Azhimov et al., 2026). Therefore, Islam mainly served as a moral and cultural guide, rather than a complete legal system regulating every facet of life.

After gaining independence, the Kazakh state emphasizes traditional Islam as a moderate, non-political, and culturally authentic form of religion that goes hand in hand with secular governance. In the government's narratives, the traditional Islam is seen as a counterbalance to Salafism, Hizb ut-Tahrir, and other transnational Islamist groups that are considered to be threats to social stability and national security (Yemelianova, 2010). From this point of view, traditional Islam is more than just a religious identity; it is also a security narrative that ties religious legitimacy to political loyalty and social stability.

After 2010, “Traditional Islam” was the main headline in local mass media which portrayed it as a native, moderate and culturally compatible form of Islam. Local religious experts and representatives of official Muslim institutions were instrumental in consolidating this narrative by portraying Traditional Islam as the normative antithesis to foreign or radical interpretations, which were regarded as socially destabilizing. Thus, the notion served not only as a descriptive term but also as a regulatory and discursive instrument for setting the boundaries of acceptable religious expression within the framework of a secular state.

At a certain stage the term “Traditional Islam” has been used by Islamic clerics to frame their discussions with Salafi version of Islam which by and large viewed the Islamization of Kazakh culture as a manifestation of non-Islamic practice. The resulting debate brought about a division of the society and even led to religious backlash. In the meantime, the advocates of Traditional Islam were trying to present a positive image of the integration of religious faith and local culture in the history that they illustrated by the religious practices in Kazakhstan that were to a great extent the result of the interaction, mediation and even syncretism between Islam and the traditional way of the local people rather than of a strict literalist interpretation of the scriptural texts. On this point, the local traditions of the country were viewed not as a contrast to Islam but rather as a part of its local expression and social role. Supporters also warned that complete rejection of cultural practices might lead to social divisions, push people toward extremism, and break down the sense of a community, thus working against both the survival of religion and social harmony (Adilbayev, 2025).

We examined the works of Kazakhstani scholars in this field and it appeared that none of them gave an unambiguous definition of “Traditional Islam.” The majority of the research conceptualize the idea from the standpoint of historical practices, cultural customs, and local religious traditions rather than formulating a precise theoretical framework. Therefore, the term is frequently employed in an intuitive way as if there were a tacit understanding between the scholars and readers. This interpretation is consistent with findings from other post-Soviet contexts, where “Traditional Islam” operates as a discursive construct shaped by state institutions and religious organizations to define legitimate religious practice and reinforce normative boundaries (Khabibullina, 2021). This absence of conceptual clarity results in inconsistency of interpretation and application in different academic pieces. Hence, “Traditional Islam” is still a broadly used but barely defined concept in Kazakhstani academic writings, thus, calling for a more systematic and analytical definition.

Nonetheless, judging by the available materials, we suggest the following definition of “Traditional Islam” in the context of Kazakhstan. It is a form of Sunni Islam traditionally prevalent in the region and deeply influenced by, local customs, social norms, and the cultural traditions of the Kazakh people. This kind of Islam is mostly linked to the Hanafi school of law and the Maturidi creed. It teaches the virtues of moderation, social unity, and respect for cultural heritage while upholding the main Islamic principles. Thus, Traditional Islam in Kazakhstan is a combination of religious teachings and native cultural practices resulting from the historical process over the centuries.

### 3.3 Evolution of Traditional Islam in Kazakhstan

The emergence of the term “Traditional Islam” in Kazakhstan is largely a result of the worldwide and regional growth of religious extremism, as well as the wars in the Middle East in the first decade of the 2000s and 2010s. As radical ideas spread across borders and new religious movements emerged, Islamic scholars, government officials, and security representatives in Kazakhstan increasingly adopted the concept of Traditional Islam in their discourse. They used it to distinguish legitimate local Islamic practices from those viewed as foreign and extremist. During the period, Kazakhstan, similarly to other post-Soviet states, witnessed a religious revival for the first time in decades after years of Soviet repression. However, this revival happened at the same time as the worldwide spread of ultraconservative trends like Salafism and the emergence of extremist groups in war-torn areas such as Iraq, Syria, and Afghanistan. The news about some young Kazakhstani people going to fight with foreign militants only exacerbated the national worries about ideological invasion and breakdown of social order.

In response, the religious experts advocated “Traditional Islam” as a protective framework sensible to the country's historical, cultural, and theological heritage. They highlighted the Hanafi Maturidi tradition, Sufi elements, and old local customs as characteristics of a kind, tolerant, and community-oriented Islamic identity. Through this story, Traditional Islam was equated with “peaceful Islam,” thus setting a clear separation from what the government officials termed “radical” or “non-traditional” Islam—ideologies which were seen as foreign, divisive, or possibly violent (Tursyn, 2025). In Turkic Muslim contexts, the discourse of “Traditional Islam” frames it as peaceful, socially integrative, and aligned with state priorities, often contrasting it with politicized or transnational Islamic movements (Bekkin, 2021).

This division had various functions. It directed public opinion by persuading people that Kazakhstan had a native, peaceful Islam tradition, different from the ones in the conflict-torn Middle East. It gave the government a theoretical basis for the policy measures to combat extremism, while at the same time, controlling religious education, mosque activities, and public discourse. On the contrary, detractors say that such a stance neglects the plural nature of Islam and may lead to the marginalization of genuine religious practices that do not conform to the officially authorized ones. The term “Traditional Islam” therefore, even though criticized in some ways, was one of the expressions of Kazakhstan's strategy aimed at maintaining social harmony, strengthening national security, and creating a stable religious identity under the fast-changing global conditions.

Development of the Traditional Islam in Kazakhstan has been additionally confused by increasing influence of global Islamic movements that, on a regular basis, promoted their alternative interpretations of faith and practice. As Kazakhstan continues to exist in its post-Soviet identity, the revival of Islamic thought has brought about a discussion between traditional values and modern beliefs, resulting in a complex religious scene that combines local traditions with the wider Islamic principles (Sadvokassov and Zhumashev, 2023). This dynamic interaction brings up very serious issues about the future of religious authority and community cohesion, especially considering that young people nowadays increasingly use digital platforms which give them access to a variety of theological perspectives. Therefore, the main problem is how to satisfactorily integrate modern influences with the historical roots of Islam in Kazakhstan so that the essential traditional practices are kept intact while at the same time being changed inevitably by the realities of a globalized world.

Eventually, such ongoing debates will determine not only the religious landscape but also the cultural character of Kazakhstan, which is trying to find a balance between its past and the future. The research presented here points to the necessity of further in-depth studies on how global influences and digital technologies affect the transformation of traditional Islamic practices in Kazakhstan. These changes include the increased use of social media in the formation of religious identity and in bringing closer traditional and contemporary modes of practice (Osmonova et al., 2024). The detailed insight into these phenomena would be very helpful for the government, educators, and religious leaders who are interested in creating a milieu where tradition goes hand in hand with modernity. Only such a transformation can be the key to the survival of Islam in its traditional form and at the same time, it can guarantee Islam's appeal to the younger generation of Kazakhstan.

### 3.4 Comparative perspectives on the construction of “Traditional Islam” in different national contexts

This section adopts a structured comparative approach to examine how different states construct and deploy the concept of “Traditional Islam.” The analysis focuses on three dimensions: (1) institutional structure, (2) definition of “Traditional Islam,” and (3) its political and social function. This framework enables a consistent comparison across national contexts and supports the argument that “Traditional Islam” operates as a state-mediated construct rather than a purely theological category.

#### 3.4.1 Russia

##### 3.4.1.1 Institutional structure

In Russia, Islam is organized through state-recognized muftiates and centralized spiritual administrations, reflecting a long-standing tradition of state-managed religious authority dating back to the imperial period (Crews, 2006). This institutional framework continues to shape contemporary governance of Islam.

##### 3.4.1.2 Definition of “Traditional Islam”

“Traditional Islam” is typically defined as a historically rooted form of Sunni Islam, primarily associated with Hanafi jurisprudence, local customs, and continuity with established religious practices, while rejecting transnational or politicized interpretations (Bekkin, 2020).

##### 3.4.1.3 State function

The concept functions as a governance tool aimed at promoting political loyalty, countering extremism, and reinforcing national identity. In this context, religious legitimacy is closely tied to alignment with state interests, illustrating how “Traditional Islam” operates as a regulatory and political category rather than a purely theological one (Bedford et al., 2021).

#### 3.4.2 Morocco

##### 3.4.2.1 Institutional structure

In Morocco, religious authority is centralized under the monarchy, where the king holds the title of Commander of the Faithful, granting direct control over religious institutions, education, and preaching (Tozy, 1999).

##### 3.4.2.2 Definition of “Traditional Islam”

Traditional Islam is defined through a combination of Maliki jurisprudence, Ash'ari theology, and Sufi spirituality, presented as the authentic religious heritage of Moroccan society.

##### 3.4.2.3 State function

This model reinforces political legitimacy and social stability, particularly in the context of countering extremism and maintaining centralized religious authority. The promotion of “Traditional Islam” thus serves both religious and governance functions.

#### 3.4.3 Egypt

##### 3.4.3.1 Institutional structure

In Egypt, al-Azhar operates as a central institution of Islamic authority, functioning in close alignment with the state while maintaining its historical status as a leading center of Sunni scholarship (Brown, 2014).

##### 3.4.3.2 Definition of “Traditional Islam”

Traditional Islam is framed as a form of moderate Sunni Islam grounded in established scholarly traditions and positioned in opposition to Islamist and Salafi movements.

##### 3.4.3.3 State function

The concept is used to legitimize state-supported religious authority and to limit alternative interpretations perceived as politically destabilizing. This reflects the increasing alignment between religious discourse and state priorities (Hallaq, 2013).

#### 3.4.4 Indonesia

##### 3.4.4.1 Institutional structure

In Indonesia, Traditional Islam is shaped through a combination of state institutions and influential civil society organizations, particularly Nahdlatul Ulama (NU), which plays a central role in religious life (Fealy and White, 2008).

##### 3.4.4.2 Definition of “Traditional Islam”

It is defined through adherence to Shafi'i jurisprudence, Ash'ari theology, and Sufi-influenced practices, emphasizing pluralism, tolerance, and cultural accommodation (Fealy and White, 2008).

##### 3.4.4.3 State function

Traditional Islam serves as a counterweight to transnational Islamist ideologies and supports a model of religious pluralism aligned with national identity and state policy (Fealy and White, 2008).

To further operationalize the comparison, the key dimensions of institutional control, discursive framing, and political function across cases are summarized in Table 1.

**Table 1:** Features of *Traditional Islam* across selected countries.

Country	Institutional control	Discursive framing	Political function
Kazakhstan	SAMK (centralized)	Hanafi–Maturidi, national tradition	Stability, national identity
Russia	State muftiates	Traditional Sunni, loyal	Integration, counter-extremism
Morocco	Monarchy	Maliki–Ash'ari–Sufi tradition	Legitimacy, stability
Egypt	Al-Azhar	Moderate Sunni	Control, legitimacy
Indonesia	State + NU	Pluralist traditional islam	Pluralism, counter-radicalism

As shown in Table 1, despite contextual variation, all cases exhibit similar patterns across institutional control, discursive framing, and political function, reinforcing the argument that “Traditional Islam” is mainly shaped by governance priorities.

Besires, across these cases, it appears that “Traditional Islam” functions less as a fixed theological category and more as a state-mediated construct shaped by governance priorities, thereby reinforcing the interpretation of the Kazakhstani case as part of a broader global pattern.

### 3.5 Problematizing “Traditional Islam” in states without an indigenous Islamic tradition

The cases we have discussed above are all from countries with a Muslim majority or states where Muslims have been present historically. But in case of states without historic Islam in this context, what does “Traditional Islam” mean? In countries where Islam came mainly through migration and not through historical continuity, Traditional Islam cannot be a concept tied to local religious practices that are hundreds of years old. On the contrary, it is more often the case that such a notion is established by the recognition of institutions, accommodation of laws and normative frameworks of the modern nation-state. Here, “tradition” is less about the original religious heritage and more about forms of Islam that are considered as compatible with civic values, secular governance, and social cohesion.

Similarly, in countries such as the United States and Canada, where Islam lacks an established historical rootedness, Traditional Islam is not defined by state doctrine but by community-led institutions that emphasize classical Sunni norms, scholarly continuity, and ethical integration within a pluralistic society. In these cases, “tradition” often refers to adherence to inherited Islamic jurisprudence and theology rather than to cultural or national specificity (Jackson, 2005).

Governments in Western Europe, for instance, often support a version of implementing “moderate” or “domesticated” Islam that, covertly, reproduces the logic of Traditional Islam in the post-Soviet world. In those situations, Traditional Islam is therefore “made” through the intervention of officially approved representative bodies, standardized training of imams, and controlled religious education, not through local historical development. Researchers point out that this practice tends to result in a so-called “nationalized” Islam that highlights allegiance to the constitutional order and keeps a certain distance from transnational religious authorities (Kaliyeva et al., 2025).

In this regard, the French approach of “Frenchization” Islam stands out as a particularly telling example. Rather than Muslims being part of the historical communities of the country, France has mainly directed Islam through legal, institutional, and civic frameworks that reflect the republican principles of secularism (laïcité), national sovereignty, and social cohesion. Frenchization is a process that changes Islam from a transnational religious identity into a domesticated religious practice that is compatible with the values of the French constitution. The centerpiece of this strategy is the government's endeavor to form Muslim institutions that can be representatives and thus be the mediators between the religious communities and the public authorities. The efforts to create the Council of the Muslim Faith in France (CFCM) and, lately, the Forum of Islam in France (FORIF) show the attempts to control religious leadership, standardize the management of mosques and limit the involvement of foreign countries in the financing of religion and the training of imams (Bowen, 2010).

Within this framework, “Traditional Islam” in France does not imply historically inherited religious practice but to a normatively acceptable form of Islam—one that is apolitical, transparent in funding, and respectful of republican norms. Tradition is thus redefined as compliance with civic expectations rather than continuity with a particular jurisprudential or theological lineage. As a result, the French model prioritizes institutional loyalty and legal conformity over classical markers of Islamic authority such as scholarly chains of transmission or adherence to established schools of law.

The French case hence is a good example illustrating the general pattern of non-historic Islamic contexts: in places where Islam is not historically entrenched, the state usually plays the main role in defining what is considered “Traditional” or “authentic” Islam. In these environments, tradition is not passed down but made, influenced by political rationality, legal norms, and worries about integration and security. This thus strengthens the point that “Traditional Islam,” is not a stable theological category but a notion that heavily depends on the context and is significantly impacted by the mechanisms of modern governance.

### 3.6 Mechanisms and scope of the promotion of Traditional Islam in Kazakhstan

The propagation of Traditional Islam in Kazakhstan operates on several levels simultaneously, intertwining the state's policy, the structures of the religious institutions, and the practices of the local communities. This multi-level faith promotion strategy illustrates a balancing act on the part of the country to maintain religious harmony, national integration and to keep away radical or non-traditional interpretations of Islam.

At the state level, Traditional Islam remains one of the main influences on Kazakhstan's historical and cultural identity and hence, it is officially recognized by the state. Also, the government policy encourages a model of Islam, which is regarded as being in line with the values of moderation, tolerance, and social harmony. In terms of organization, the major instrument of Traditional Islam in Kazakhstan is the Spiritual Administration of Muslims of Kazakhstan which is a unified body of all Muslims of Kazakhstan. It administrates the mosques, imam appointments, religious authorities, and theological consistencies of the teachings across the country. Religious sermons, publications, and different types of seminars emphasize good behavior, respect for the values and customs of the nation, and loyalty to the state. This indicates that religious authority is not decentralized but institutionally monopolized, allowing the state to define the boundaries of legitimate Islam.

Mass media play a very important role in the dissemination of the process as well. Television programs, online platforms and printed materials produced under government control present Traditional Islam as an inseparable part of Kazakh history and culture. They offer religious knowledge in a simple form and strive to dismantle extremism use and misinformation. On a grassroots level, Traditional Islam is mainly supported through daily religious practices, family rituals, and life-cycle ceremonies such as weddings, funerals, and anniversaries. These activities perpetuate a culturally ingrained type of Islam which, rather than strict dogmatism, emphasizes good moral behavior, social responsibility, and communal fellowship. The older generation, local religious leaders, and family traditions are the main channels for propagating these values over generations (Urynbai, 2021). Social networks are very helpful in spreading religion in the digital space as they make it very easy to share religious teachings, sermons, and educational content with a large number of people quickly. This implies broader transformations in n the age of media, where institutional actors increasingly shape religious knowledge through controlled communication channels (Eickelman and Anderson, 2003). Apart from that, they allow religious scholars and followers to communicate, creating virtual communities and dialogues that go beyond traditional church settings. On the other hand, these media also frame the religious narratives, thus impacting the way people interpret and live their faith in the modern world (Kerim et al., 2025).

SAMK media (websites and official social media pages) are the main avenue of Traditional Islam promotion in Kazakhstan. The thematic analysis of SAMK media outputs reveals a set of recurring discursive patterns that structure the representation of “Traditional Islam” in official communication. Four dominant themes emerge consistently across the analyzed materials: (1) the construction of Islam as inherently moderate and tolerant, (2) its alignment with national traditions and cultural identity, (3) its association with social stability and political order, and (4) the boundary-making distinction between “traditional” and “non-traditional” Islam. The consistency of these themes across different media formats indicates a structured process of discursive standardization rather than isolated messaging, suggesting that official religious communication functions as a mechanism for defining and regulating legitimate Islam (Kurmanaliyev et al., 2025).

To examine SAMK preaching activity empirically, this study conducted a qualitative thematic analysis of media outputs produced by SAMK. The dataset includes 52 items (sermons, website articles, and social media posts) published on official SAMK platforms between January 2024 and November 2025. Materials were selected as representative forms of official religious communication. Inductive thematic approach was used to identify recurring discursive patterns, guided by the study's analytical framework.

The analysis reveals four dominant themes. First, Islam is framed as inherently moderate:

“*Islam is a religion of moderate path*.” (Article, September 2025)

Second, it is linked to national identity:

“*Our ancestors followed Islam in harmony with Kazakh traditions*” (Article, March 2024)

Third, it is associated with social stability and civic loyalty:

“*A true Muslim contributes to peace and respects the laws of the country*.” (Sermon, March 2024)

Fourth, discourse constructs boundaries between “traditional” and “non-traditional” Islam:

“*Anything that contradict our traditional path must be rejected*.” (Sermon, May 2024)

The consistency of these themes across platforms indicates a structured and standardized discourse, suggesting that SAMK media function as a mechanism for defining and regulating legitimate Islam in alignment with state priorities.

### 3.7 Current trends of Traditional Islam in Kazakhstan

In Kazakhstan, Traditional Islam continues to play a significant role in shaping cultural identity, social values, and the broader religious landscape of the country. Predominantly following the Sunni Hanafi school with strong historical and cultural roots, Islam in Kazakhstan is marked by its tolerance, adaptability, and integration within a multi-confessional society.

After collapse of the Soviet Union, religious freedoms expanded, leading to a revival of Islamic institutions, education, and public religious life. Mosques, religious organizations, and spiritual centers have grown, reflecting both an increase in self-identified Muslims and community participation in religious education and practice (Malgarayeva, 2024). One of the notable trends is the institutional strengthening of Traditional Islam, particularly under the leadership of the Spiritual Association of Muslims of Kazakhstan. The organization permanently advocate for interfaith harmony, social stability, and moral values rooted in the Kazakh cultural context.

Contemporary discourse also highlights a renewed focus on family and ethical values as central to traditional Islamic teaching. Religious leaders have publicly discussed the importance of reinforcing family structures and cultural norms amid globalizing influences, often framing this as part of preserving national identity. Moreover, there is a growing interest in Islamic education and literacy, especially among youth. Increased attendance at mosques, enrollment in religious study programs, and public engagement with Islamic teachings point to a trend of seeking deeper understanding of faith beliefs—while the state works alongside religious institutions to counteract radical ideologies and promote peaceful, tolerant interpretations of Islam (Akhmetkali, 2025). Kazakhstan's model of religious development reflects a deliberate balance between secular governance and recognition of Islam's role in cultural heritage. Traditional Islam is not only a faith practice but also contributes to national dialogues on ethics, identity, and social cohesion in a modern, diverse society.

### 3.8 Prospects and challenges for Traditional Islam

One major prospect for Traditional Islam is its role in supporting social cohesion and national identity. Researchers note that traditional Islam influences integrative processes within Kazakh society, helping shape national identity and promote tolerance amid ethnic and religious diversity (Mukan et al., 2023). The institutional presence of the Spiritual Administration of Muslims of Kazakhstan (SAMK) fosters moderate religious practice, promotes interfaith dialogue, and contributes to social stability.

Traditional Islam also has the potential to counteract radical interpretations by offering a rooted, culturally relevant framework for understanding faith that resonates with local believers. Experts argue that emphasizing classical Islamic scholarship and traditional madhhab (jurisprudence) can serve as a bulwark against imported extremist ideas.

Despite these prospects, there are significant challenges. One key issue is the influence of non-traditional religious interpretations entering Kazakhstan through global media and foreign preaching. These alternative ideologies can confuse believers and sometimes compete with the country's historically moderate religious norms.

Another challenge is maintaining interfaith and interethnic harmony. As Islam's visibility grows, there are occasional tensions with other religious communities, which underscores the need for inclusive dialogue and policies that uphold secular governance alongside religious freedom. The future of Traditional Islam in Kazakhstan involves strengthening its positive role in society through education, cultural engagement, and interfaith cooperation, while addressing challenges linked to external ideological influences.

## 4 Conclusions

This study has demonstrated that “Traditional Islam” in Kazakhstan is best understood as a dynamic, historically layered, and politically mediated theological phenomenon. While Islamic practices rooted in Hanafi jurisprudence, Maturidi creed, Sufi ethics, and local customs have existed on the Kazakh steppe for centuries, the contemporary use of the term “Traditional Islam” reflects modern challenges associated with post-Soviet transformation, globalization, and state-led religious governance.

Historically, Islam in Kazakhstan developed as a flexible and adaptive tradition embedded in nomadic social life and customary law. Its emphasis on moral conduct, communal solidarity, and cultural continuity enabled Islamic identity to survive periods of imperial domination and Soviet repression. The Soviet era, despite systematic secularization policies, did not eliminate Islam but transformed it into a largely cultural and private form of religiosity. This legacy significantly shaped the post-independence religious revival, which was driven more by cultural memory than by formal theological education.

In the post-1991 period, the concept of Traditional Islam gained renewed prominence as the Kazakhstani state sought to regulate religious life and prevent the spread of radical ideologies. By promoting a model of Islam grounded in the Hanafi–Maturidi tradition and aligned with national customs, state institutions positioned Traditional Islam as both an authentic expression of Kazakh heritage and a safeguard of social stability. This process reflects a broader pattern observed across post-Soviet and Muslim-majority contexts, where “tradition” is defined through institutional frameworks and security considerations rather than purely religious criteria.

The comparative analysis conducted in this study further illustrates that Traditional Islam functions as a state-centered discourse in many national settings. Whether in Russia, Morocco, Egypt, or parts of Western Europe, governments invoke tradition to legitimize certain religious interpretations while marginalizing others deemed politically or socially undesirable. In this sense, Traditional Islam serves governance objectives by linking religious authenticity to loyalty, moderation, and conformity with state norms. While such an approach may contribute to stability, it also risks narrowing religious plurality and conflating theological diversity with security threats.

One of the key findings of this research is the persistent lack of conceptual clarity surrounding Traditional Islam in Kazakhstani scholarship. Most studies describe the phenomenon empirically without offering a systematic theoretical definition. This ambiguity allows the term to function flexibly in public discourse but also creates analytical inconsistencies. By proposing a working definition of Traditional Islam as a historically established form of Sunni Islam shaped through interaction with local culture and institutional regulation, this study seeks to address this gap and provide a clearer analytical foundation for future research.

Looking forward, Traditional Islam in Kazakhstan faces both opportunities and challenges. Its cultural rootedness and ethical orientation position it as a potential source of social cohesion and interreligious tolerance. At the same time, globalization, digital media, alternative religious ideologies, and competing identity narratives—such as the revival of pre-Islamic belief systems—pose challenges to its authority and relevance, particularly among younger generations. Addressing these challenges requires not only institutional control but also inclusive education, open dialogue, and scholarly engagement with the diversity of Islamic expression.

From a theoretical perspective, the findings of this study support Asad's argument that religion is not a self-evident or purely theological category but one shaped through historical and political processes. The case of Kazakhstan demonstrates how “Traditional Islam” is constructed through institutional regulation and state discourse. At the same time, the analysis reflects Bourdieu's insight that religious authority is produced through relations of symbolic power, where dominant institutions define orthodoxy and regulate legitimacy. In this sense, “Traditional Islam” emerges not simply as a religious tradition but as a product of state-mediated power and governance.

This study produces several key findings. First, it demonstrates that “Traditional Islam” in Kazakhstan functions not merely as a theological or historical category, but as a state-mediated construct shaped through institutional control, discursive framing, and regulatory practices. Second, the analysis shows that official religious discourse, particularly through SAMK media, systematically constructs a normative model of Islam aligned with state priorities such as social stability, cultural continuity, and political loyalty. Third, the study reveals that the boundaries of “Traditional Islam” are actively maintained through discursive exclusion of alternative interpretations labeled as “non-traditional” or “foreign.” These findings contribute to understanding how states shape religious legitimacy in contemporary contexts.

However, state-centered promotion of “Traditional Islam” raises important critical questions. The centralization of religious authority in state-aligned institutions such as SAMK may limit pluralism in the Islamic field, reducing space for alternative interpretations and independent religious voices. This institutional monopolization can marginalize groups that do not conform to officially sanctioned norms, reinforcing distinctions between “acceptable” and “non-traditional” Islam. Moreover, such top-down approaches may not fully resonate with younger generations, who increasingly engage with diverse religious perspectives through digital platforms beyond state control. Tensions may therefore emerge between officially promoted religious narratives and the lived religious experiences of believers. While the model of “Traditional Islam” contributes to stability and governance, it may also generate unintended social and ideological frictions that require further critical examination.

In conclusion, Traditional Islam in Kazakhstan should be understood as an evolving synthesis of faith, culture, and governance rather than theological phenomenon. The nuanced and critical approach to this concept is essential for advancing academic debates, informing balanced religious policy, and preserving social cohesion within an increasingly complex religious landscape.

## Data Availability

The original contributions presented in the study are included in the article/supplementary material, further inquiries can be directed to the corresponding author.

## Appendix

### Definitions of Islamic terms used in the article

**Hanafi**: one of the four major Sunni Islamic schools of jurisprudence, known for its emphasis on reason, analogy (*qiyās*), and flexibility in legal interpretation. **Maturidi**: school of Sunni Islamic theology that emphasizes rationality and human free will, commonly associated with regions following the Hanafi tradition. **madrasa**: Islamic educational institution where religious sciences, including theology and law, are taught. **adat**: local customary law and social practices that coexist with or complement Islamic law in many Muslim societies. **Shari'a**: comprehensive body of Islamic law derived from the Qur'an and Sunnah, governing religious, moral, and social conduct. **istihsan**: principle in Islamic jurisprudence allowing jurists to prefer equitable solutions over strict analogical reasoning when necessary. **urf**: recognized local customs or practices that can be incorporated into Islamic legal rulings if not in conflict with core principles. **Sunnah**: practices, sayings, and actions of the Prophet Muhammad, serving as a primary source of Islamic guidance. **madhhab**: recognized school of Islamic jurisprudence that provides methodologies for interpreting and applying Islamic law. **Maliki**: Sunni school of Islamic law that places strong emphasis on the practices of the people of Medina as a source of legal authority. **Ash'ari**: major school of Sunni theology that seeks a balance between revelation and rational thought, often associated with mainstream orthodoxy. **Shafi'i**: Sunni legal school that systematized the principles of Islamic jurisprudence, particularly emphasizing hadith as a source of law. **Nahdlatul Ulama (NU)**: large Indonesian Islamic organization promoting traditional Sunni Islam, pluralism, and moderate religious values. **Hizb ut-Tahrir**: transnational Islamist political organization advocating for the re-establishment of a global Islamic caliphate.
